# Profiling and bioinformatics analyses of differential circular RNA expression in prostate cancer cells

**DOI:** 10.4155/fsoa-2018-0046

**Published:** 2018-10-03

**Authors:** Chunlei Zhang, Jun Xiong, Qi Yang, Ye Wang, Haoqing Shi, Qinqin Tian, Hai Huang, Depei Kong, Jianmin Lv, Dan Liu, Xu Gao, Xiaoyuan Zi, Yinghao Sun

**Affiliations:** 1 Department of Urology, Changhai Hospital, Second Military Medical University, Shanghai, PR China; 2 Department of Urology, Lanzhou General Hospital of PLA, Lanzhou, PR China; 3 Department of Histological Embryology, Second Military Medical University, Shanghai, PR China; 4 Department of Cell Biology, Second Military Medical University, Shanghai, PR China

**Keywords:** bioinformatics analyses, circular RNA, high-throughput sequencing, noncoding RNA, prostate cancer

## Abstract

**Aim:**

There is little knowledge about the expression profile and function of circular RNAs (circRNAs) in prostate cancer (PCa).

**Methods:**

The expression profiles of circRNAs in RWPE-1, 22RV1 and PC3 cells were explored via high-throughput circRNAs sequencing and validated by real-time qPCR. The roles of differentially expressed circRNAs were evaluated by bioinformatics analyses.

**Results:**

Altogether 9545 circRNAs were identified and hundreds of differentially expressed circRNAs were recognized. CircRNA–miRNA networks analysis showed that many circRNAs, including circSLC7A6, circGUCY1A2 and circZFP57 could cross-talk with tumor-related miRNAs such as miR-21, miR-143 and miR-200 family.

**Conclusion:**

The results of our bioinformatics analyses suggested that circRNAs should play critical roles in the development and progression of PCa.

## Lay abstract

The expression profile and role of circular RNAs (circRNAs) in prostate cancer (PCa) is unknown. In this study, the profiling of circRNAs in PCa cells was explored and their potential function was predicted by bioinformatics analyses. In total, 9545 circRNAs and hundreds of differentially expressed circRNAs were identified. Bioinformatics analyses showed that circRNAs including circSLC7A6, circGUCY1A2 and circZFP57 could cross talk with tumor-related miRNAs. This indicated that circRNAs might play an important role in PCa and warrant further study.

## Graphical abstract

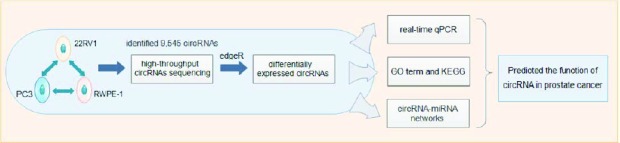

Circular RNAs (circRNAs) are naturally endogenous RNAs classically formed from pre-mRNAs by reverse splicing. They are involved in various cellular and disease processes through modulating gene transcription and post-transcription [1]. They were first discovered in viruses and thought to be caused by mis-splicing of gene sequences. With the development of high-throughput sequencing and bioinformatics analyses in recent years, thousands of circRNAs have been identified in most organisms including bacteria, plants and animals [2]. CircRNAs are evolutionarily conserved, stabilized and more enriched in exosomes [3–5]. Although they are considered as a new class of noncoding RNAs (ncRNAs), new studies have discovered that circRNAs can be translated [6,7]. More importantly, circRNAs have a species-specific, tissue-specific and cell-specific expression pattern [2], suggesting that circRNAs may play important biological roles and possess the potential to become a predictive and therapeutic target for diseases.

Although knowledge about the biological function of circRNAs is limited, some circRNAs have been found to be associated with diabetes [8], myocardial infarction [9], osteoarthritis [10] and Alzheimer's disease [11]. CircRNAs have also been demonstrated to be involved in the tumorigenesis and progression of esophageal cancer [12], hepatocellular carcinoma [13], gastric cancer [14], colon cancer [15], bladder cancer [16], ovarian cancer [17] and lung cancer [18]. For instance, circMTO1 suppressed the progression of hepatocellular carcinoma by sponging miR-9 [13], circPVT1 can be seen as a proliferative factor and prognostic marker in gastric cancer [14], and the circTCF25-miR-103a-3p/miR-107-*CDK6* pathway was reported to play an important regulatory role in bladder carcinoma [16].

Prostate cancer (PCa) is the most common malignant tumor of the male reproductive system and the second most lethal tumor disease for men [19]. A growing number of studies have demonstrated that the aberrant expression of ncRNAs contributes to cell proliferation, metastasis and drug resistance in PCa. A variety of lncRNAs and miRNAs such as PCA3, PCAT-1, MALAT1, miR-205 and miR-34a, have been experimentally and clinically reported to be involved in PCa and serve as potential biomarkers or therapeutic targets for PCa [20]. However, the expression profile and role of circRNAs remain elusive.

To clarify the expression profile and role of circRNAs in PCa, we used PCa cell lines as disease models for sequencing, a circRNA-specific sequencing method to enrich circRNAs, and normal prostate epithelial RWPE-1 cells, PCa epithelial 22RV1 cells and bone metastatic cells of PC3 PCa cell line to explore the expression profiles of circRNAs using high-throughput circRNA sequencing. The workflow is shown in Supplementary Figure 1. To the best of our knowledge, this is the first work to investigate circRNA expression profiles in these three PCa cells. It is our hope that the findings of the present study could help clarify the roles of circRNAs in different stages of PCa progression and provide a promising candidate for the diagnosis and therapeutics of PCa.

## Methods

### Cell culture

Cell culture: RWPE-1, 22RV1 and PC3 cells were obtained from the American Type Culture Collection. RWPE-1 cells were cultured in PEpiCM (ScienCell, CA, USA); 22RV1 cells were cultured in RPMI-1640 (Gibco, MD, USA) with 10% fetal bovine serum and 1% penicillin–streptomycin solution; and PC3 cells were cultured in F-12 (Ham) (Gibco) with 10% fetal bovine serum and 1% penicillin–streptomycin solution, all at 37°C in a humidified incubator containing 5% CO_2_. The medium was replaced every 2 days, and cells were digested at room temperature with 0.5 ml 0.25% trypsin/EDTA (Gibco) per well when they grew to 70–80% confluence.

### RNA extraction

Total RNA was isolated from cells with Trizol reagent (Invitrogen, MA, USA) according to the manufacturer's protocol. The RNA concentration of each sample was measured by Nanodrop 2000 (Thermo Scientific, MA, USA). RNA integrity and gDNA contamination were measured by denaturing agarose gel electrophoresis. To remove residual genomic DNA, each RNA sample was digested with Rnase-free DNase I (Takara, Beijing, PR China) according to the manufacturer's instruction.

### RNA library preparation & sequencing

CircRNA-seq service and the following bioinformatic analyses were performed by CloudSeq Biotech Inc. (Shanghai, PR China). For each sample, 5 μg of total RNA was incubated for 15 min at 37°C with 3 units μg^-1^ of RNase R (Epicentre, WI, USA) to enrich circular RNA. The RNase-R treated RNA was then rRNA depleted by using the Ribo-Zero Magnetic Gold Kit (Epicentre). The rRNA-depleted RNA was used to construct the RNA libraries with TruSeq Stranded Total RNA Library Prep Kit (Illumina, CA, USA) according to the manufacturer's instructions. The library quality was evaluated with BioAnalyzer 2100 system (Agilent Technologies, CA, USA). Library sequencing was performed on an illumina Hiseq instrument with 150 bp paired end reads.

### RNA-seq data analysis

The FASTQ reads were aligned to the human reference genome. Transcriptome data were obtained from the UCSC genome database by using the BWA-MEM software [21], circRNAs were identified by using CIRI software as described [22] and the identified circRNAs were then annotated with circDB database, a manually curated database that collected all published circular RNAs and was developed by Cloudseq Inc. (For each sample, the original back-spliced junction reads of each circRNA were converted to tags per million reads.) Then, statistical hypothesis based on binomial distribution was used to identify the differentially expressed circRNAs between two sample groups by edgeR analysis method. p-value < 0.05 was considered as significantly differential expression [23,24]. Hierarchical clusterings were performed by using the euclidean distance matrix of the heatmap.2 function in the gplots package of the R environment to analyze the expression pattern of significantly up and downregulated circRNAs in each group with the threshold of fold change 2.0 and p-value < 0.05. The function of circRNAs was predicted by Gene Ontology (GO) term and KEGG pathway enrichment analyses of the corresponding host genes with DAVID database. The results of GO term and Kyoto Encyclopedia of Genes and Genomes (KEGG) pathway enrichment analyses were shown with threshold p < 0.05 and arrangement in order based on false discovery rate (FDR) value.

### circRNA–miRNA regulatory network

The significantly differentially expressed circRNAs were validated with real time qPCR, and then subjected to analysis. Differentially expressed circRNAs were used to predict the potential miRNA response elements and the binding sites of miRNAs with CloudSeq's home-made software based on miRanda and TargetScan (CloudSeq Inc.). The network between circRNAs and miRNAs was also constructed by Cytoscape based on the binding sites of the differentially expressed circRNAs and miRNAs. Different nodes represent circRNAs and miRNAs, respectively. Solid lines between two nodes represent potential binding.

### Real-time qPCR

cDNA was synthesized from 500 ng total RNA with the PrimeScript RT Master Mix (Takara), according to manufacturer's instructions. The real-time PCR analyses of the expression level of the circRNAs were performed by using SYBR Premix Ex Taq II (Takara). RT-qPCR was performed in 20 μl reaction volume, including 1 μl cDNA, 10 μl 2 × Master Mix, 0.3 μl Forward Primer (10 μM), 0.3 μl Reverse Primer (10 μM) and 8.4 μl double distilled water. The primer sequences are presented in Supplementary Table 3. The reaction was set at 95°C for 10 min, and then repeated for 40 cycles at 95°C for 10 s and 60°C for 60 s in real time PCR System (ABI, CA, USA), using β-actin as a reference. All samples were amplified in triplicate wells, and the relative level was calculated with 2^-△△Ct^ method.

### Polymerase chain reaction

The expression levels of the circRNAs were analyzed by PCR using Premix Taq (Ex Taq II) (Takara) in 20 μl reaction volume, including 1 μl cDNA, 10 μl 2 × master mix, 0.3 μl forward primer (10 μM), 0.3 μl reverse primer (10 μM) and 8.4 μl double distilled water. The reaction was set at 95°C for 10 min, repeated for 40 cycles at 95°C for 20 s, 60°C for 30 s, 72°C for 45 s and finally 72°C for 7 min in PCR System (BIO-RAD, CA, USA).

### Agarose gel electrophoresis

The products of real-time qPCR or PCR were validated in agarose (Biowest, Spain) with a concentration of 1.5% in TAE buffer (made up of Tris base, acetic acid, and EDTA; Songon Biotech, Shanghai, PR China) by use of an electrophoresis system (BIO-RAD, CA, USA). The results were detected by using an imaging system (Syngene, Cambridge, UK).

### Sanger sequencing

The PCR amplified products were Sanger sequenced by standard methods in the Beijing Genomics Institute (Beijing, PR China).

## Results

### Overview of circRNA profiles in PCa cell lines

**Figure 1.**
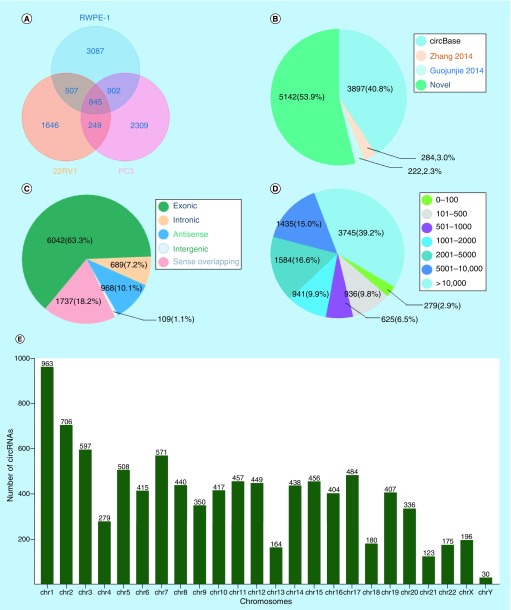
**Circular RNAs expression profiles in prostate cancer cell lines.** **(A)** The number of circRNAs identified in RWPE-1, 22RV1, PC3 cell lines. **(B)** The distribution of circRNAs from different database resources. **(C)** The distribution of circRNAs from different classifications based on the genomic origin. **(D)** The distribution of circRNAs in different lengths. **(E)** The distribution of circRNAs in different chromosomes. circRNA: Circular RNA.

To explore the profile of circRNAs in prostate carcinogenesis, biological triplicates, RNase R digested and rRNA depleted, circRNA-specific sequencing was performed in RWPE-1, 22RV1 and PC3 cells. More than 20 million raw reads on average were detected in each cell sample and more than 90% of reads were mapped (Supplementary Table 1). The number of circRNAs decreased with the increase of the mean junction reads in each cell sample (Supplementary Figure 2). A total of 9545 circRNAs were identified in the three cell lines, and 845 of them were coexpressed (Figure 1A). In these circRNAs, 53.9% (5142/9545) were novel, and the others (4403/9545, 46.1%) were annotated from Circbase database, Zhang 2014 [25] and Guojunjie 2014 [4] reports (Figure 1B). These circRNAs aligned to ‘exonic’ (6042/9545), ‘intronic’ (689/9545), ‘antisense’ (968/9545), ‘intergenic’ (109/9545) and others (1737/9545), of which exon-derived circRNAs were the most (63.3%) (Figure 1C). Meanwhile, they ranged in length from 17 to 499,794 nt, and the longest length group (>10,000 nt) accounted for the largest proportion (3745/9545, 39.2%) (Figure 1D). The distribution of host genes in chromosomes was also significantly different, most (963/9545, 10.4%) in chromosome 1 and least (30/9545, 0.31%) in chromosome Y (Figure 1E). In addition, many host genes could produce a diversity of circRNAs as a result of variable splicing, and the maximum number of variable splices was as great as 34 (Supplementary Figure 3).

### Identification of differentially expressed circRNA profiles

**Figure 2.**
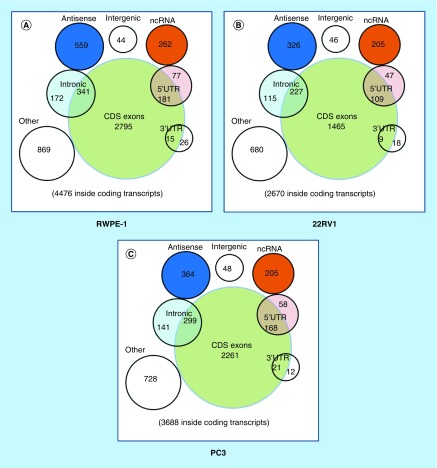

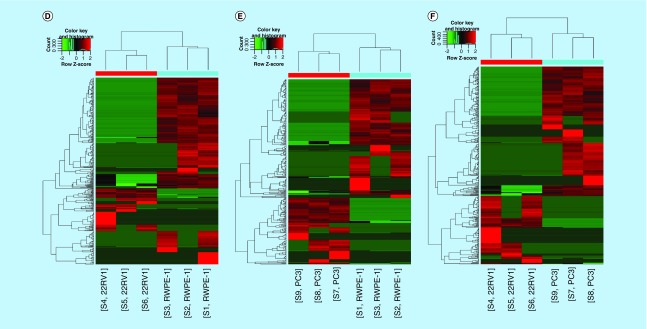

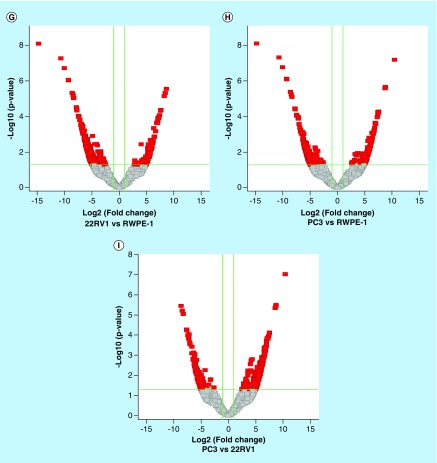
**Profiles of differentially expressed circular RNAs in prostate cancer cell lines.** **(A–C)** The distribution of circRNAs from different catalogs in RWPE-1, 22RV1 and PC3 cell lines. **(D–F)** Hierarchical clusterings shown as the analysis of differential circRNAs in group 22RV1 versus RWPE-1, PC3 versus RWPE-1, PC3 versus 22RV1, where the red strips represent high relative expression, the green strips represent low relative expression and the dendrograms show the relationships between the samples and differential circRNAs, with the threshold of fold change 2.0 and p-value < 0.05. **(G–I)** The visualization of circRNAs between two conditions in group 22RV1 versus RWPE-1, PC3 versus RWPE-1, PC3 versus 22RV1, where the red rectangles represent differential expression of circRNAs with the threshold of fold change 2.0 and p-value < 0.05.

To identify the differentially expressed circRNAs, we used statistical analysis in the groups of 22RV1 versus RWPE-1, PC3 versus RWPE-1, PC3 versus 22RV1. Venn diagrams showed the distribution of the three cell lines in each catalog and these circRNAs were mainly exon-derived and antisense-derived (Figure 2A–C). The expression pattern of hierarchical clusterings was distinguishable between different cell samples, and the dendrograms showed relationships between the samples and differential circRNAs (Figure 2D–F). Volcano plots were used to find out the target circRNAs in each group with p-value < 0.05, fold change >2.0 (Figure 2G–I) combining with scatter plots (Supplementary Figure 4A–C). A total of 443 (101 up- and 342 down-regulated) circRNAs were identified in the group of 22RV1 versus RWPE-1; 362 (130 up- and 232 down-regulated) circRNAs were identified in the group of PC3 versus RWPE-1; and 409 (268 up- and 141 down-regulated) circRNAs were identified in the group of PC3 versus 22RV1. All the differentially expressed circRNAs are shown in Supplementary Table 2. We enumerated the 10 significantly up and downregulated circRNAs in each group (Table 1). The distribution of the differentially expressed circRNAs derived from different chromosomes and catalogs are shown in Table 2. Most host genes of differentially expressed circRNAs were still exon-derived and in chromosome 1.

**Table 1.** **Significantly upregulated and downregulated differentially expressed circular RNAs.**

**Name**	**Groups**	**CircRNAID**	**Log_2_FC**	**Chrom**	**CircBaseID**	**Catalog**	**GeneName**	**Predicted sequence length**
CircGUCY1A2	22RV1 vs RWPE-1	chr11:106849345|106856857	8.40	chr11	hsa_circ_0008602	Exonic	*GUCY1A2*	184

CircETV3	22RV1 vs RWPE-1	chr1:157068933|157104019	7.05	chr1		Sense overlapping	*ETV3*	35,087

CircKCNN2	22RV1 vs RWPE-1	chr5:113740135|113740553	6.01	chr5		Exonic	*KCNN2*	419

CircMIR663A	22RV1 vs RWPE-1	chr20:26189806|26190011	6.42	chr20		Intronic	*MIR663A*	206

CircBAGE2	22RV1 vs RWPE-1	chr21:11038728|11058323	5.79	chr21	hsa_circ_0061259	Exonic	*BAGE2*	1152

CircKRT6A	22RV1 vs RWPE-1	chr12:52841323|52881739	-10.04	chr12		Sense overlapping	*KRT6A*	40,417

CircCD276	22RV1 vs RWPE-1	chr15:73995113|73996338	-7.82	chr15		Exonic	*CD276*	654

CircZFP57	22RV1 vs RWPE-1	chr6:29643163|29643836	-7.44	chr6		Exonic	*ZFP57*	229

CircPSMA7	22RV1 vs RWPE-1	chr20:60714131|60716000	-7.36	chr20	hsa_circ_0003456	Exonic	*PSMA7*	375

CircRPPH1	22RV1 vs RWPE-1	chr14:20811283|20811436	-7.21	chr14	hsa_circ_0000512	Sense overlapping	*RPPH1*	154

CDR1as	PC3 vs RWPE-1	chrX:139865340|139866824	7.55	chrX	hsa_circ_0001946	Antisense	*CDR1*	1485

CircDPF3	PC3 vs RWPE-1	chr14:73181131|73198642	6.97	chr14		Exonic	*DPF3*	303

CircPRKG1	PC3 vs RWPE-1	chr10:54048486|54050050	6.81	chr10		Exonic	*PRKG1*	253

CircSLCO4A1	PC3 vs RWPE-1	chr20:61291764|61292527	6.69	chr20		Exonic	*SLCO4A1*	234

CircETV3	PC3 vs RWPE-1	chr1:157068933|157104019	5.77	chr1		Sense overlapping	*ETV3*	35,087

CircKRT6A	PC3 vs RWPE-1	chr12:52841323|52881739	-10.04	chr12		Sense overlapping	*KRT6A*	40,417

CircRPPH1	PC3 vs RWPE-1	chr14:20811405|20811559	-7.45	chr14		Sense overlapping	*RPPH1*	155

CircZFP57	PC3 vs RWPE-1	chr6:29643163|29643836	-7.44	chr6		Exonic	*ZFP57*	229

CircEMB	PC3 vs RWPE-1	chr5:49694941|49707217	-7.17	chr5	hsa_circ_0001481	Sense overlapping	*EMB*	12,277

CircFKBP5	PC3 vs RWPE-1	chr6:35586873|35610620	-7.13	chr6	hsa_circ_0001599	Exonic	*FKBP5*	527

CDR1as	PC3 vs 22RV1	chrX:139865340|139866824	7.55	chrX	hsa_circ_0001946	Antisense	*CDR1*	1485

CircASPH	PC3 vs 22RV1	chr8:62593523|62596747	7.39	chr8		Sense overlapping	*ASPH*	3225

CircMTCL1	PC3 vs 22RV1	chr18:8718422|8720494	7.13	chr18	hsa_circ_0000825	Exonic	*MTCL1*	384

CircDPF3	PC3 vs 22RV1	chr14:73181131|73198642	6.97	chr14		Exonic	*DPF3*	303

CircSLC7A6	PC3 vs 22RV1	chr16:68300496|68300624	3.13	chr16	hsa_circ_0039943	Exonic	*SLC7A6*	129

CircGUCY1A2	PC3 vs 22RV1	chr11:106849345|106856857	-8.40	chr11	hsa_circ_0008602	Exonic	*GUCY1A2*	184

CircMAN1A2	PC3 vs 22RV1	chr1:117944808|117984947	-6.64	chr1	hsa_circ_0000119	Exonic	*MAN1A2*	648

CircMIR663A	PC3 vs 22RV1	chr20:26189806|26190011	-6.42	chr20		Intronic	*MIR663A*	206

CircKCNN2	PC3vs 22RV1	chr5:113740135|113740553	-6.01	chr5		Exonic	*KCNN2*	419

CircEMB	PC3 vs 22RV1	chr5:49694941|49707217	-5.76	chr5	hsa_circ_0001481	Sense overlapping	*EMB*	12,277

Name: The names of cirRNAs appearing in this article designated by their host genes; CircRNAID: The ID of the identified circRNA by CIRI (to distinguish from canonical mRNA transcripts, the coordinate positions for each circRNA are connected with a vertical bar ‘|’ instead of a dash ‘-’); Log_2_FC: Logarithm of the fold change of normalized reads between two groups of samples; Chrom: The chromosome in which the circRNA lies; CircBaseID, the identifier of circBase (www.circbase.org). GeneName: The name of the circRNA-associated gene (best transcript); Catalog: The catalog of the host genes, including ‘exonic’, ‘intronic’, ‘antisense’, ‘intergenic’ and ‘sence overlapping’; Predicted sequence length: The length of predicted circRNA sequence.

**Table 2.** **Distribution of differentially expressed circular RNAs in each chromosome and catalog.**

**Chromosome**	**Up.22RV1 vs RWPE-1**	**Down.22RV1 vs RWPE-1**	**Up.PC3 vs RWPE-1**	**Down.PC3 vs RWPE-1**	**Up.PC3 vs 22RV1**	**Down.PC3 vs 22RV1**
chr1	11	31	15	28	18	14

chr2	5	26	7	11	20	8

chr3	4	21	8	12	21	4

chr4	4	8	3	6	5	4

chr5	2	34	2	18	14	8

chr6	5	11	5	14	9	11

chr7	12	14	8	5	15	14

chr8	8	14	12	10	24	9

chr9	1	16	2	14	8	9

chr10	2	7	12	8	14	5

chr11	3	19	2	13	11	4

chr12	3	8	5	8	8	7

chr13	0	0	1	2	1	2

chr14	12	28	18	18	30	9

chr15	5	21	0	16	9	7

chr16	7	7	1	8	3	7

chr17	2	19	12	13	18	4

chr18	1	3	3	2	5	2

chr19	4	10	4	4	14	5

chr20	4	28	2	12	9	3

chr21	1	3	3	1	3	0

chr22	2	8	1	2	2	1

chrX	3	6	3	4	6	3

chrY	0	0	1	3	1	1

Exonic	44	240	66	128	184	78

Antisense	3	22	7	25	19	10

Intronic	11	19	13	24	11	14

Intergenic	1	5	2	6	2	2

Sense overlapping	42	56	42	49	52	37

### Validation of the differentially expressed circRNAs in PCa cells

**Figure 3.**
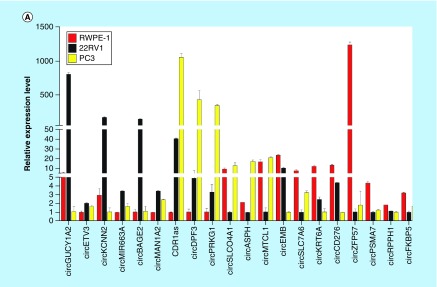

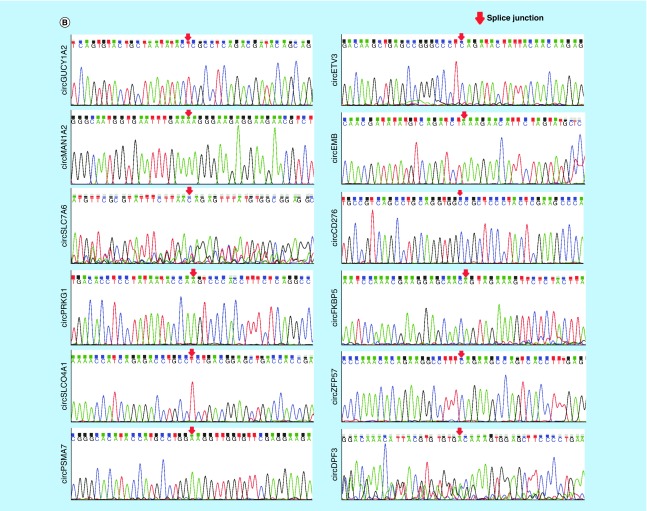

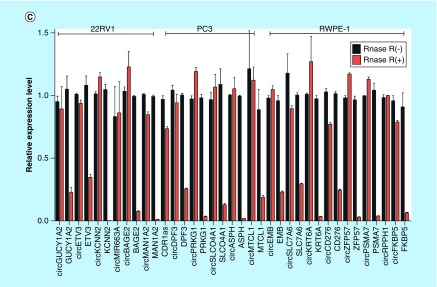
**Validation of differentially expressed circular RNAs in prostate cancer cell lines.** **(A)** The relative expression levels of significantly up- and down-regulated circRNAs in RWPE-1, 22RV1 and PC3 cell lines by real-time qPCR. **(B)** The Sanger sequencing results of 12 circRNAs PCR amplified products selected randomly. **(C)** Real-time qPCR results of circRNAs in PCa cells treated with RNase R. The amount of circRNAs was normalized to the value measured in the mock treatment. circRNA: Circular RNA.

To validate the high-throughput sequencing data, the expression of up and downregulated circRNAs in RWPE-1, 22RV1, PC3 cell samples was verified by real-time qPCR (Figure 3A), and the results were confirmed by agarose gel electrophoresis (Supplementary Figure 5). Expression detected by each of the two methods was consistent with each other, demonstrating the high reliability of the RNAseq results. The PCR amplified products of 12 deregulated circRNAs chose randomly were confirmed by Sanger sequencing (Figure 3B). To verify the characteristics of stability, circRNAs were treated with RNase R. As expected, the circRNAs were resistant to RNase R treatment in contrast to linear control (Figure 3C).

### Prediction of circRNA functions in PCa

To further explore the functions of circRNAs in the biological progression of PCa, we performed GO term and KEGG pathway analyses on the host genes of differentially expressed circRNAs.

**Figure 4.**
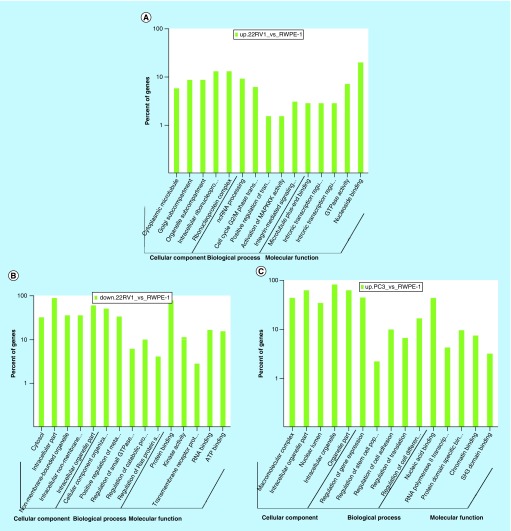

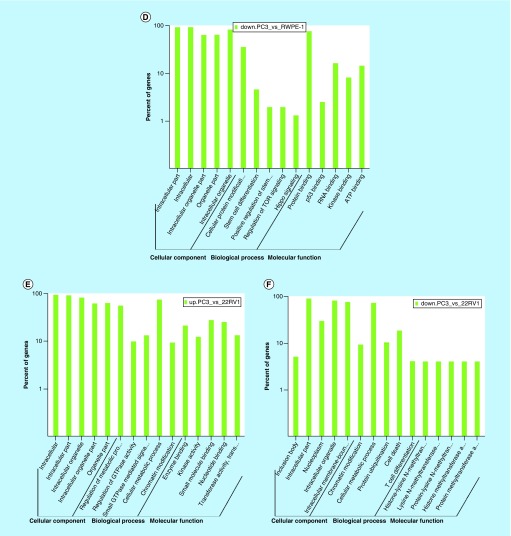

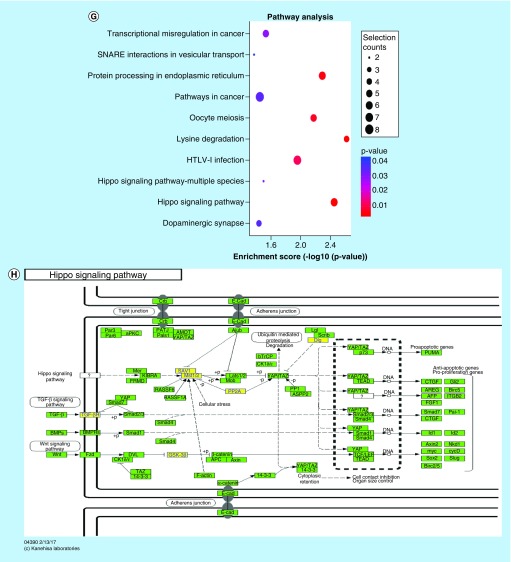

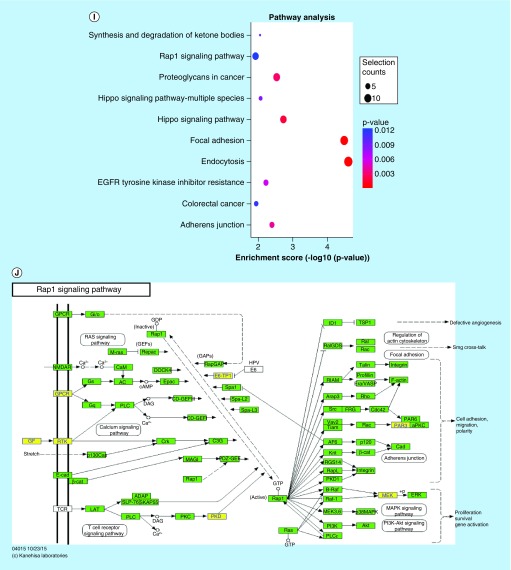
**Gene ontology term analysis and KEGG pathway analysis of differentially expressed circular RNA host genes.** **(A–F)** Main GO term analysis of 22RV1 versus RWPE-1, PC3 versus RWPE-1 and PC3 versus 22RV1 with p-value < 0.05 and enrichment score > 1.0; the values plotted on Y axis are the percentage of the corresponding host genes in total genes of each group. **(G, I)** Top 10 pathways shown by KEGG pathway analysis in the group PC3 versus RWPE-1 (downregulation) and 22RV1 versus RWPE-1 (downregulation) with p-value < 0.05 and enrichment score > 1.0. **(H, J)** The pathway maps of ‘Hippo signaling pathway’ and ‘Rap1 signaling pathway’, where the yellow-marked nodes are host genes associated with downregulated circRNAs and the green nodes have no significance.

All comparison data of GO term analyses including  biological process, molecular function and cellular component are shown in Supplementary Figure 6, 7 & 8. Our biological process analyses showed that some host genes could regulate the molecular metabolism process, catabolic process, cell cycle G2/M phase transition, small GTPase mediated signal transduction and the characteristics of stem cells. Some other host genes were involved in regulation of translation, protein ubiquitination, kinase activity and signaling pathways, such as integrin-mediated signaling, TOR signaling pathway, Hippo signaling pathway and Ras protein signal transduction. Molecular function analyses showed that host genes had the ability to bind many kinds of molecules, including SH3 domains, p53, enzymes, DNA, RNA and ATP (Figure 4A–F).

KEGG pathway revealed that some host genes were associated with cancer progression by participating in proteoglycan and transcriptional misregulation. In addition, the analyses also showed that some genes had enrichment on a variety of tumor-related signaling pathways (including Hippo, Rap1, ErbB, Wnt, PI3K-Akt, HIF-1, Notch and TGF-β signaling pathways). All comparison data are shown in Supplementary Figure 9. The significantly important pathways with high enrichment scores were ‘Hippo signaling pathway’ in the group of PC3 versus RWPE-1 (downregulation) and ‘Rap1 signaling pathway’ in the group of 22RV1 versus RWPE-1 (downregulation) (Figure 4G–J).

### Construction of circRNA–miRNA interaction networks

**Figure 5.**
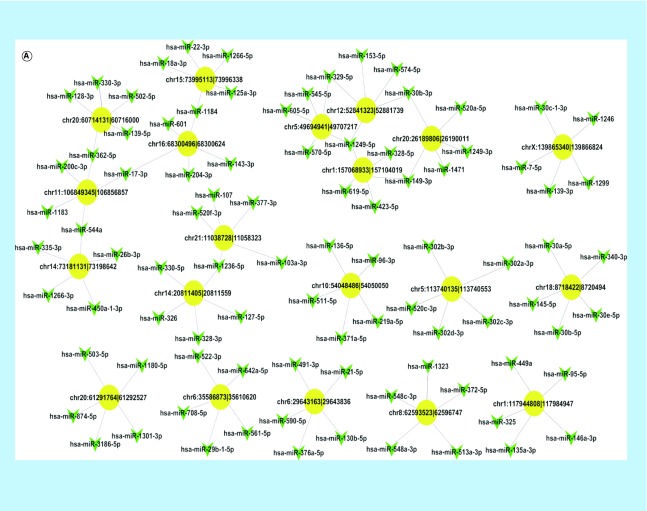

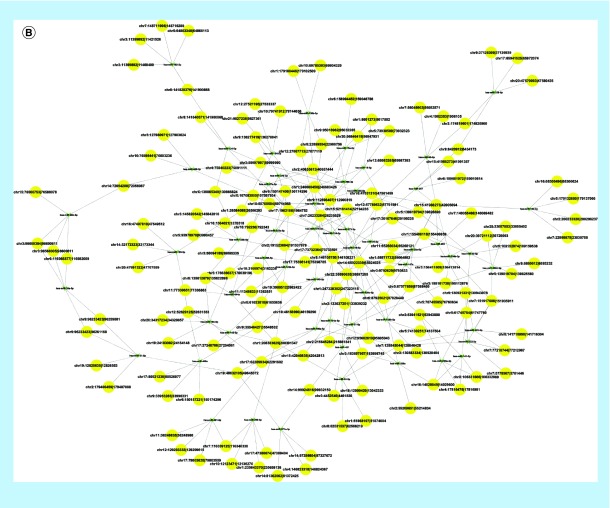
**The predicted circRNA–miRNA interaction networks.** **(A)** The circRNA–miRNA network of differentially expressed circRNAs and five miRNAs with stronger binding ability, where the yellow-marked nodes are circRNAs and the green marked nodes are miRNAs. **(B)** The circRNA–miRNA network of 46 miRNAs closely related to the development of prostate cancer with the corresponding differentially expressed circRNAs. circRNA: Circular RNA.

To illustrate the complex regulation mechanism of circRNAs in PCa cells, we constructed two circRNA–miRNA networks. First, we constructed a circRNA–miRNA network with differentially expressed circRNAs listed above and the five miRNAs with the stronger binding ability to them (Figure 5A). To find out the target circRNAs associated with PCa more effectively, we selected 46 miRNAs closely related to the development of PCa (including miR-21, miR-221/222 and the miR-200 family) to draw a network diagram for showing the relationship between them and the corresponding circRNAs (Figure 5B).

## Discussion

CircRNAs are natural endogenous RNAs, the roles of which, in some human cancers, have been gradually unraveled in recent years [1]. However, the role of circRNAs in PCa is not clear yet. In the present study, we reported the circRNA expression profiles in PC3, 22RV1 and RWPE-1 cell lines and explored the differentially expressed circRNAs to predict the potential function of circRNAs in PCa by bioinformatics analyses. Unlike mRNA participating in signaling of the biological process by translating into protein, circRNAs can regulate some important biological activities via different mechanisms, such as regulating the content of miRNAs as a reservoir of miRNAs [2], combining with protein to form a circRNA-protein complex to disrupt the effects of protein on the targets [26], regulating host gene transcription by binding with RNA polymerase II or miRNA [27] and affecting the formation of gene splicing protein [28]. They can also be translated into protein directly in a few cases [6,7].

The most important mechanism of circRNAs is known to function as miRNA sponges like competitive endogenous RNA  molecules [2]. miRNAs are important regulators in gene expression and play a crucial role in cancer progression. In this study, we constructed circRNA–miRNA networks by using bioinformatics analyses to explore the relationship between differentially expressed circRNAs and miRNAs. Some important miRNAs were found to bind with the significantly differential circRNAs listed above, such as miR-143 (binding with circSLC7A6, hsa_circ_0039943), miR200c and miR-200b (binding with circGUCY1A2, hsa_circ_0008602) and miR-21 (binding with circZFP57). According to previous studies, miR-143 was associated with bone metastasis of PCa and involved in the regulation of epithelial-to-mesenchymal transition [29]; the miR-200 family could inhibit PCa progression [30]; and miR-21 could promote PCa development [31], implying that circGUCY1A2 and circZFP57 may affect the development of tumor formation and circSLC7A6 may promote bone metastasis through these miRNAs. The selected 46 miRNAs, which were reported to be closely associated with PCa progression [30–34], or with AR signaling pathway [35–40] and epithetlial-to-mesenchymal transition [29,41–45], could also be found to have corresponding circRNAs. These results indicate that circRNAs may have a close relationship with the development of PCa.

Another important mechanism of circRNAs is that they can affect the expression or function of host genes. GO term analyses of this study showed that host genes of the differentially expressed circRNAs functioned in some important biological processes and molecular mechanisms, such as binding of the SH3 domain and participating in integrin-mediated signaling pathway. DOCK1, for example, has a SH3-SH2-domain in LNCap cells, and ROBO1 interacts with DOCK1 to negatively regulate the Rac activation at this domain [46]. FUT8 provides a versatile *N*-glycosylated protein for the formation of *N*-glycan branches. *N*-glycan is a basic and generic protein modification in mammals and plays a key role in various physiological and pathological events including cancer progression [47]. Branch variations are often found in cancer cells and are deeply involved in cancer growth, invasion and metastasis. In addition, hundreds of host genes such as *DOCK1*, *FUT8*, *MPP6*,  *GSK3B*, *SETD3* are capable of binding with proteins, and therefore further study is warranted to see whether the corresponding circRNAs of these host genes would function by binding with protein.

KEGG pathway analysis of this study demonstrated that many host genes were involved in Hippo, Rap1, ErbB, Wnt, PI3K-Akt, HIF-1, Notch and TGF-β signaling pathways, all of which are known as important mechanisms for cancer progression. The Hippo signaling pathway, also known as the Salvador/Warts/Hippo pathway, is an evolutionarily conserved pathway that controls cell proliferation, apoptosis and stem cell self-renewal [48]. Its main function is phosphorylating the main modulation proteins, YAP and TAZ, in this pathway [49]. Once dephosphorylated, the coactivator will translocate to the nucleus and promote the expression of genes that contribute toward cell proliferation and survival, which in turn accelerates cancer progression [50]. Through Hippo signaling, Wnt signaling promotes androgen-independent prostate cancer cell proliferation [51], α3β1 integrin suppresses prostate cancer metastasis [52], and phosphodiesterase 5/protein kinase G signal governs stemness of prostate cancer stem cells [53]. Rap1, a small GTPase in the Ras-related protein family, is regulated by binding to GTP or GDP and functions as a switch during cellular signaling transduction, which has many important effects in tumor cell migration, invasion and metastasis [54]. In PCa, Rap1 signaling mainly regulates PCa cell adhesion and promotes PCa metastasis [55,56].

In the present study, we detected the differential expression of circRNAs in PCa cells. CDR1as (hsa_circ_0001946) was validated to highly express in PC3. CDR1as is a circRNA containing 74 binding sites of miR-7, and its typical ‘miRNA Sponge’ mechanism has been studied in depth [2]. The downstream genes of miR-7 include *KLF4*, *RAF1*, *PAK1*, *IRS2*, *IRS1*, *AKT*, *ACK1*, *FAK*, *IGF1R*, *HNF4*, *SNCA*, *mTOR*, *NOTCH1* and *PIK3CD* [57]. All of these are indispensable tumor suppressors or oncogenes in tumorigenesis of various cancers. In the sequencing results, the expression of CDR1as in PC3 was nearly 200 times higher than that in normal prostate epithelial cells and prostate carcinoma epithelial cells, suggesting that CDR1as may substantially contribute to bone metastatic progress of PCa. CircBAGE2 (hsa_circ_0061259) is significantly upregulated in 22RV1 compared with RWPE-1. The binding miRNA miR-103a can suppress tumor cell proliferation by targeting PDCD10 in PCa [58], suggesting that circBAGE2 may have a close relevance with the progression of PCa.

## Conclusion

From the data above, we conclude that circular RNAs may have a close relationship with the development and progression of PCa. CircRNAs such as circGUCY1A2, circZFP57 and circBAGE2 may play an important role in pathogenesis, and CDR1as and circSLC7A6 may be associated with bone metastasis. All in all, the present study revealed a comprehensive expression and functional profile of differentially expressed circRNAs in PCa cell lines, which may provide a new direction for the study of PCa.

## Future perspective

With deeper research into this field, the function of circRNAs has been paid more and more attention. The current study shows that the expression of circRNAs in PCa cells is definitely abundant and bioinformatics analyses also predict that circRNAs may play an important role in PCa. However, the profile of differentially expressed circRNAs in the specimens of PCa patients is unknown, and the real tumor biology functions of circRNAs in PCa remain elusive. These should be explored in the future. The interactions between mRNAs, miRNAs and circRNAs constitutes a huge competitive endogenous RNA system network, the regulatory mechanism of which also needs to be further explored.

**Summary points**

- Circular RNAs (CircRNAs) are involved in many important biological processes, as well as the pathogenesis of many cancers. The role of circRNAs in prostate cancer (PCa) is unknown.
- The current study aimed to explore the profile of circRNAs in PCa cell lines and to predict their potential function.
- In total, 9545 circRNAs and hundreds differentially expressed circRNAs were identified in PCa cell lines.
- CircGUCY1A2, circZFP57 and circBAGE2 should be important in the pathogenesis of PCa.
- CDR1as and circSLC7A6 should be associated with bone metastasis in PCa.
- The profiling and differentially expressed circRNAs in the specimens of PCa patients should be explored in the future.

## References

[1] Jeck WR, Sorrentino JA, Wang K (2013). Circular RNAs are abundant, conserved, and associated with ALU repeats. *RNA*.

[2] Memczak S, Jens M, Elefsinioti A (2013). Circular RNAs are a large class of animal RNAs with regulatory potency. *Nature*.

[3] Chen W, Schuman E (2016). Circular RNAs in brain and other tissues: a functional enigma. *Trends Neurosci.*.

[4] Guo JU, Agarwal V, Guo H, Bartel DP (2014). Expanded identification and characterization of mammalian circular RNAs. *Genome Biol.*.

[5] Lasda E, Parker R (2016). Circular RNAs co-precipitate with extracellular vesicles: a possible mechanism for circRNA clearance. *PLoS ONE*.

[6] Legnini I, Di Timoteo G, Rossi F (2017). Circ-ZNF609 is a circular RNA that can be translated and functions in myogenesis. *Mol. Cell*.

[7] Pamudurti NR, Bartok O, Jens M (2017). Translation of circRNAs. *Mol. Cell*.

[8] Xu H, Guo S, Li W, Yu P (2015). The circular RNA Cdr1as, via miR-7 and its targets, regulates insulin transcription and secretion in islet cells. *Sci. Rep.*.

[9] Geng HH, Li R, Su YM (2016). The circular RNA Cdr1as promotes myocardial infarction by mediating the regulation of miR-7a on its target genes expression. *PLoS ONE*.

[10] Liu Q, Zhang X, Hu X (2016). Circular RNA related to the chondrocyte ECM regulates MMP13 expression by functioning as a MiR-136 ‘sponge’ in human cartilage degradation. *Sci. Rep.*.

[11] Lukiw WJ (2013). Circular RNA (circRNA) in Alzheimer's disease (AD). *Front. Genet.*.

[12] Xia W, Qiu M, Chen R (2016). Circular RNA has_circ_0067934 is upregulated in esophageal squamous cell carcinoma and promoted proliferation. *Sci. Rep.*.

[13] Han D, Li J, Wang H (2017). Circular RNA circMTO1 acts as the sponge of microRNA-9 to suppress hepatocellular carcinoma progression. *Hepatology*.

[14] Chen J, Li Y, Zheng Q (2017). Circular RNA profile identifies circPVT1 as a proliferative factor and prognostic marker in gastric cancer. *Cancer Lett.*.

[15] Hsiao KY, Lin YC, Gupta SK (2017). Noncoding effects of circular RNA CCDC66 promote colon cancer growth and metastasis. *Cancer Res.*.

[16] Zhong Z, Lv M, Chen J (2016). Screening differential circular RNA expression profiles reveals the regulatory role of circTCF25-miR-103a-3p/miR-107-CDK6 pathway in bladder carcinoma. *Sci. Rep.*.

[17] Ahmed I, Karedath T, Andrews SS (2016). Altered expression pattern of circular RNAs in primary and metastatic sites of epithelial ovarian carcinoma. *Oncotarget*.

[18] Zhu X, Wang X, Wei S (2017). hsa_circ_0013958: a circular RNA and potential novel biomarker for lung adenocarcinoma. *FEBS J.*.

[19] Center MM, Jemal A, Lortet-Tieulent J (2012). International variation in prostate cancer incidence and mortality rates. *Eur. Urol.*.

[20] Ma G, Tang M, Wu Y, Xu X, Pan F, Xu R (2016). LncRNAs and miRNAs: potential biomarkers and therapeutic targets for prostate cancer. *Am. J. Transl. Res.*.

[21] Li H, Durbin R (2009). Fast and accurate short read alignment with Burrows-Wheeler transform. *Bioinformatics*.

[22] Gao Y, Wang J, Zhao F (2015). CIRI: an efficient and unbiased algorithm for *de novo* circular RNA identification. *Genome Biol.*.

[23] Zhang X, Yan Y, Lei X (2017). Circular RNA alterations are involved in resistance to avian leukosis virus subgroup-J-induced tumor formation in chickens. *Oncotarget*.

[24] Su H, Lin F, Deng X (2016). Profiling and bioinformatics analyses reveal differential circular RNA expression in radioresistant esophageal cancer cells. *J. Transl. Med.*.

[25] Zhang XO, Wang HB, Zhang Y, Lu X, Chen LL, Yang L (2014). Complementary sequence-mediated exon circularization. *Cell*.

[26] Du WW, Yang W, Liu E, Yang Z, Dhaliwal P, Yang BB (2016). Foxo3 circular RNA retards cell cycle progression via forming ternary complexes with p21 and CDK2. *Nucleic Acids Res.*.

[27] Zhang Y, Zhang XO, Chen T (2013). Circular intronic long noncoding RNAs. *Mol. Cell*.

[28] Ashwal-Fluss R, Meyer M, Pamudurti NR (2014). circRNA biogenesis competes with pre-mRNA splicing. *Mol. Cell*.

[29] Peng X, Guo W, Liu T (2011). Identification of miRs-143 and -145 that is associated with bone metastasis of prostate cancer and involved in the regulation of EMT. *PLoS ONE*.

[30] Josson S, Chung LW, Gururajan M (2015). microRNAs and prostate cancer. *Adv. Exp. Med. Biol.*.

[31] Qin W, Zhao B, Shi Y, Yao C, Jin L, Jin Y (2009). BMPRII is a direct target of miR-21. *Acta Biochim. Biophys. Sin. (Shanghai)*.

[32] Shi XB, Xue L, Yang J (2007). An androgen-regulated miRNA suppresses Bak1 expression and induces androgen-independent growth of prostate cancer cells. *Proc. Natl Acad. Sci. USA*.

[33] Galardi S, Mercatelli N, Giorda E (2007). miR-221 and miR-222 expression affects the proliferation potential of human prostate carcinoma cell lines by targeting p27Kip1. *J. Biol. Chem.*.

[34] Calin GA, Sevignani C, Dumitru CD (2004). Human microRNA genes are frequently located at fragile sites and genomic regions involved in cancers. *Proc. Natl Acad. Sci. USA*.

[35] Ostling P, Leivonen SK, Aakula A (2011). Systematic analysis of microRNAs targeting the androgen receptor in prostate cancer cells. *Cancer Res.*.

[36] Shi XB, Xue L, Ma AH (2013). Tumor suppressive miR-124 targets androgen receptor and inhibits proliferation of prostate cancer cells. *Oncogene*.

[37] Qu F, Cui X, Hong Y (2013). MicroRNA-185 suppresses proliferation, invasion, migration, and tumorigenicity of human prostate cancer cells through targeting androgen receptor. *Mol. Cell Biochem*.

[38] Boll K, Reiche K, Kasack K (2013). MiR-130a, miR-203 and miR-205 jointly repress key oncogenic pathways and are downregulated in prostate carcinoma. *Oncogene*.

[39] Hagman Z, Haflidadottir BS, Ceder JA (2013). miR-205 negatively regulates the androgen receptor and is associated with adverse outcome of prostate cancer patients. *Br. J. Cancer*.

[40] Fendler A, Stephan C, Yousef GM, Jung K (2011). MicroRNAs as regulators of signal transduction in urological tumors. *Clin. Chem.*.

[41] Gandellini P, Folini M, Longoni N (2009). miR-205 exerts tumor-suppressive functions in human prostate through down-regulation of protein kinase Cepsilon. *Cancer Res.*.

[42] Zhu C, Li J, Cheng G (2013). miR-154 inhibits EMT by targeting HMGA2 in prostate cancer cells. *Mol. Cell. Biochem.*.

[43] Ren D, Wang M, Guo W (2013). Wild-type p53 suppresses the epithelial-mesenchymal transition and stemness in PC-3 prostate cancer cells by modulating miR145. *Int. J. Oncol.*.

[44] Ishteiwy RA, Ward TM, Dykxhoorn DM, Burnstein KL (2012). The microRNA -23b/-27b cluster suppresses the metastatic phenotype of castration-resistant prostate cancer cells. *PLoS ONE*.

[45] Qu Y, Li WC, Hellem MR (2013). MiR-182 and miR-203 induce mesenchymal to epithelial transition and self-sufficiency of growth signals via repressing SNAI2 in prostate cells. *Int. J. Cancer*.

[46] Parray A, Siddique HR, Kuriger JK (2014). ROBO1, a tumor suppressor and critical molecular barrier for localized tumor cells to acquire invasive phenotype: study in African–American and Caucasian prostate cancer models. *Int. J. Cancer*.

[47] Kizuka Y, Taniguchi N (2016). Enzymes for *N*-glycan branching and their genetic and nongenetic regulation in cancer. *Biomolecules*.

[48] Zhao B, Tumaneng K, Guan KL (2011). The Hippo pathway in organ size control, tissue regeneration and stem cell self-renewal. *Nat. Cell Biol.*.

[49] Zanconato F, Cordenonsi M, Piccolo S (2016). YAP/TAZ at the roots of cancer. *Cancer Cell*.

[50] Lamar JM, Stern P, Liu H, Schindler JW, Jiang ZG, Hynes RO (2012). The Hippo pathway target, YAP, promotes metastasis through its TEAD-interaction domain. *Proc. Natl Acad. Sci. USA*.

[51] Seo WI, Park S, Gwak J (2017). Wnt signaling promotes androgen-independent prostate cancer cell proliferation through up-regulation of the hippo pathway effector YAP. *Biochem. Biophys. Res. Commun.*.

[52] Varzavand A, Hacker W, Ma D (2016). alpha3beta1 integrin suppresses prostate cancer metastasis via regulation of the Hippo pathway. *Cancer Res.*.

[53] Liu N, Mei L, Fan X (2016). Phosphodiesterase 5/protein kinase G signal governs stemness of prostate cancer stem cells through Hippo pathway. *Cancer Lett.*.

[54] Zhang YL, Wang RC, Cheng K, Ring BZ, Su L (2017). Roles of Rap1 signaling in tumor cell migration and invasion. *Cancer Biol. Med.*.

[55] Peak JC, Jones NP, Hobbs S, Katan M, Eccles SA (2008). Phospholipase C gamma 1 regulates the Rap GEF1-Rap1 signalling axis in the control of human prostate carcinoma cell adhesion. *Oncogene*.

[56] Bailey CL, Kelly P, Casey PJ (2009). Activation of Rap1 promotes prostate cancer metastasis. *Cancer Res.*.

[57] Hansen TB, Kjems J, Damgaard CK (2013). Circular RNA and miR-7 in cancer. *Cancer Res.*.

[58] Fu X, Zhang W, Su Y, Lu L, Wang D, Wang H (2016). MicroRNA-103 suppresses tumor cell proliferation by targeting PDCD10 in prostate cancer. *Prostate*.

