# Supplementary material for: Profiling and bioinformatics analyses of differential circular RNA expression in prostate cancer cells
Source: Future Sci OA. 2018 Oct 3;4(9):FSOA340. doi: 10.4155/fsoa-2018-0046 (PMC6222276; doi:10.4155/fsoa-2018-0046)
Supplement: Supplementary file 1 [file fsoa-04-340-s1.docx]

**Supplementary information**

**Figure S1. Work flow of circRNA profiling in PCa cell lines**

**Figure S2. The distribution of circRNAs sorted by the number of average junction reads in each cell line.**

**Figure S3. The distribution of circRNAs** **sorted by the number of variable splicing isoforms.**

**Figure S4.** **Scatter plots**. **(A**-**C)** Scatter plots show the differentially expressed circRNAs in group of 22RV1 *vs.* RWPE-1, PC3 *vs.* RWPE-1, PC3 *vs.* 22RV1 respectively, red dots indicate up-regulation, and green dots indicate down-regulation, the fold change threshold is 2.0.

**Figure S5. Agarose gel electrophoresis results of differentially expressed circRNAs validation.**

**Figure S6. Go term analysis of the host genes of differentially expressed circRNAs in the group of 22RV1 *vs.* RWPE-1. (A**-**I)** Upregulation of 22RV1 *vs.* RWPE-1, **(J**-**R)** Downregulation of 22RV1 *vs.* RWPE-1.

**Figure S7. Go term analysis of the host genes of differentially expressed circRNAs in the group of PC3 *vs.* RWPE-1. (A**-**I)** Upregulation of PC3 *vs.* RWPE-1, **(J**-**R)** Downregulation of PC3 *vs.* RWPE-1.

**Figure S8. Go term analysis of the host genes of differentially expressed circRNAs in the group of PC3 *vs.* 22RV1.** **(A**-**I)** Upregulation of PC3 *vs.* 22RV1, **(J**-**R)** Downregulation of PC3 *vs.* 22RV1.

**Figure S9.** **KEGG pathway analysis of the host genes of differentially expressed circRNAs in group of 22RV1 *vs.* RWPE-1, PC3 *vs.* RWPE-1 and PC3 *vs.* 22RV1. (A)** Top 10 pathways in upregulation group of 22RV1 *vs.* RWPE-1, **(B)** Top 10 pathways in downregulation group of 22RV1 *vs.* RWPE-1, **(C)** Top 10 pathways in upregulation group of PC3 *vs.* RWPE-1, **(D)** Top 10 pathways in downregulation group of PC3 *vs.* RWPE-1, **(E)** Top 10 pathways in upregulation group of PC3 *vs.* 22RV1, **(F)** Top 10 pathways in downregulation group of PC3 *vs.* 22RV1.

**Table S1. Reads statistics.** Note：**S1,S2,S3**：RWPE-1 cell lines samples; **S4,S5,S6:** 22RV1 cell lines samples; **S7,S8,S9**：PC3 cell lines samples.

**Table S2. The differentially expressed circRNAs in each group.**

**Table S3. Primers in the validation of differentially expressed circRNAs.**

**Figure S1**

**
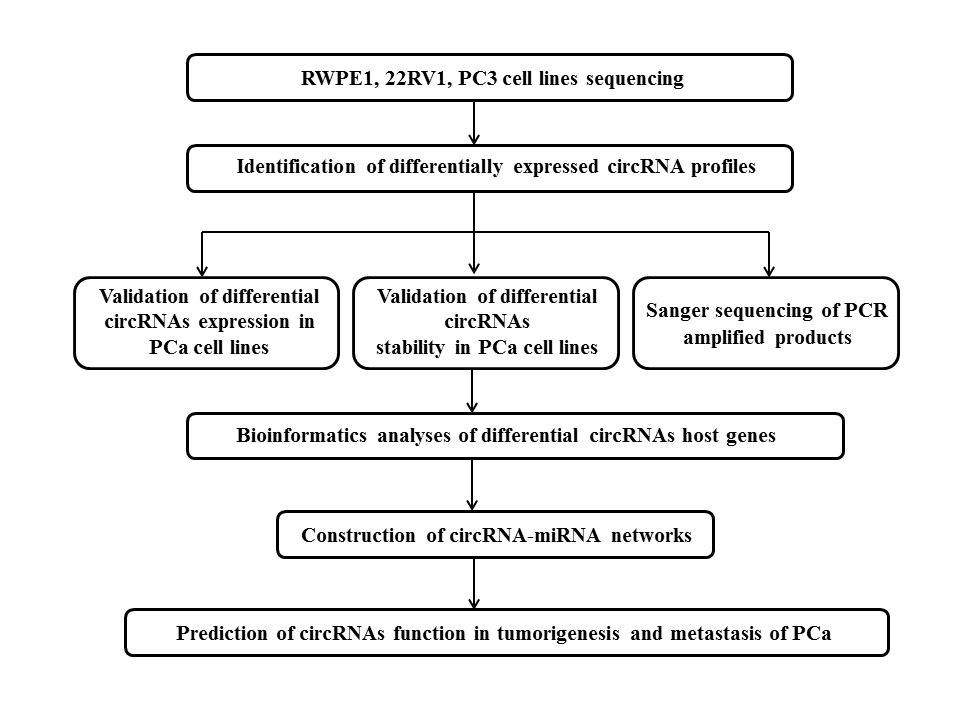
**

**Supplementary Figure S2**


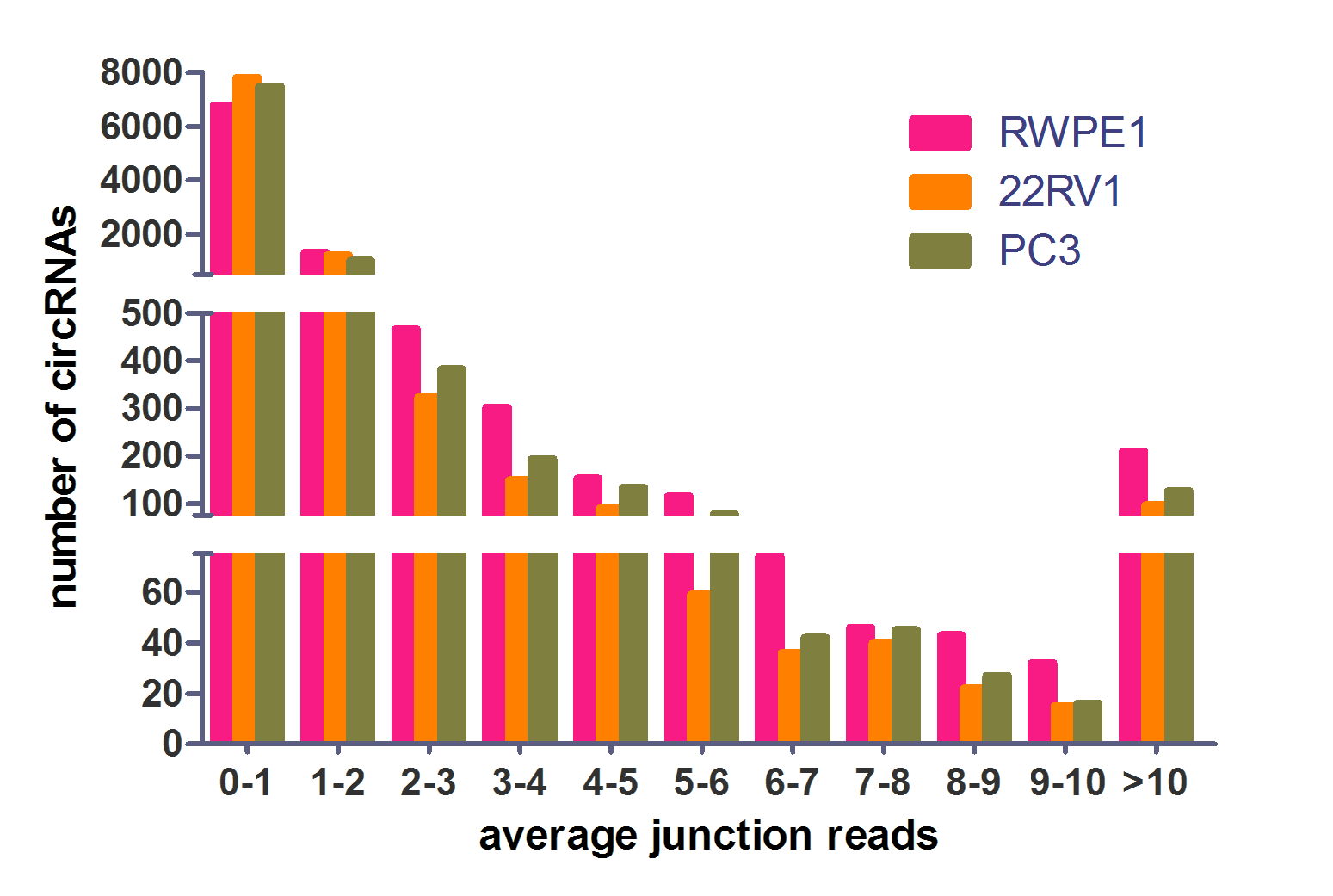


**Supplementary Figure S3**


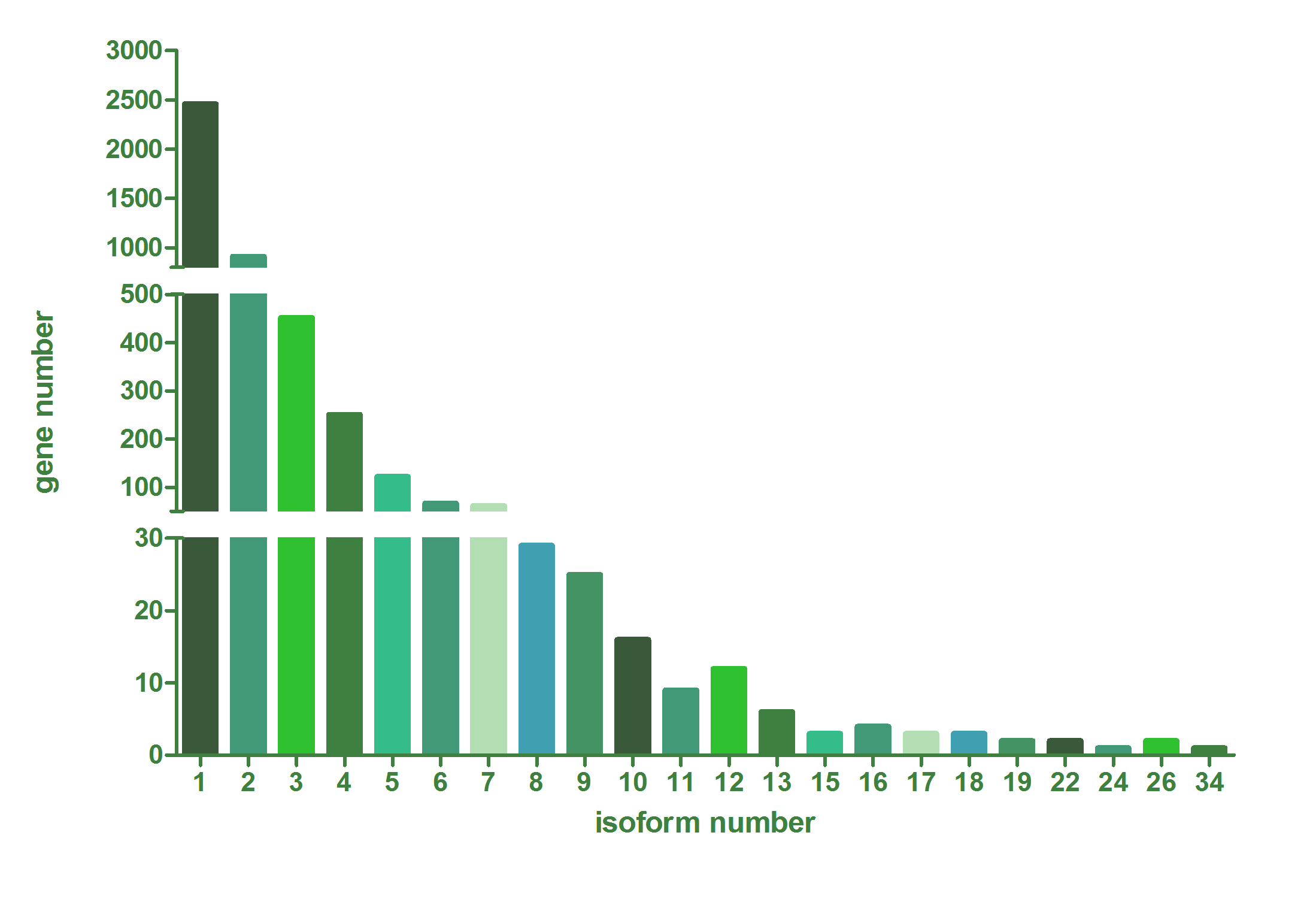


**Supplementary Figure S4**


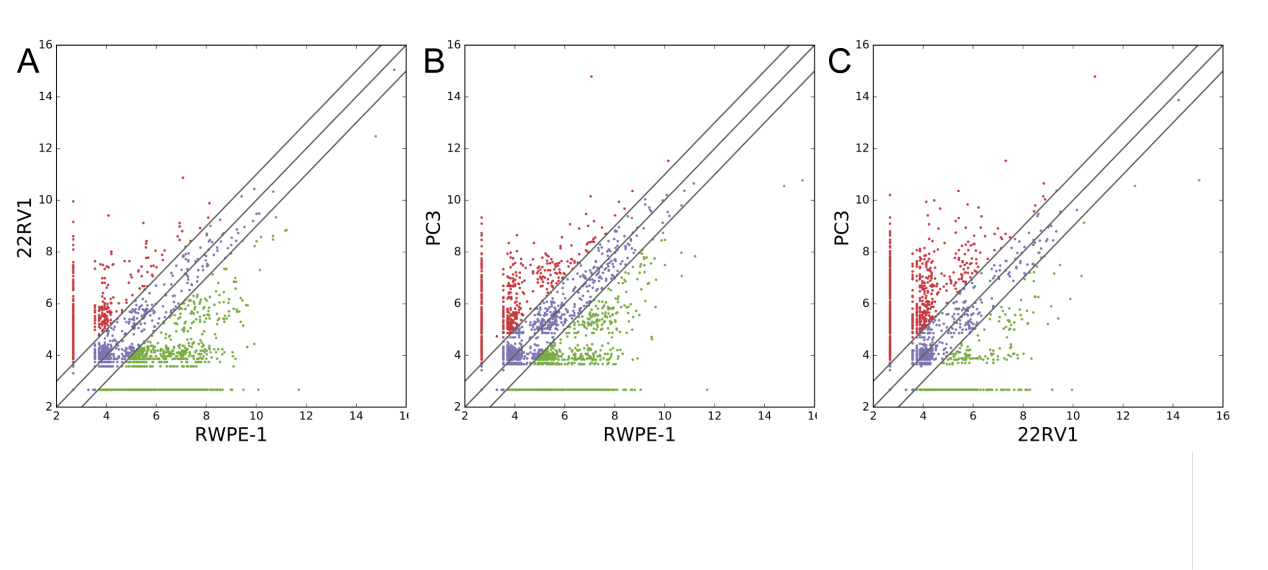


**Supplementary Figure S5**


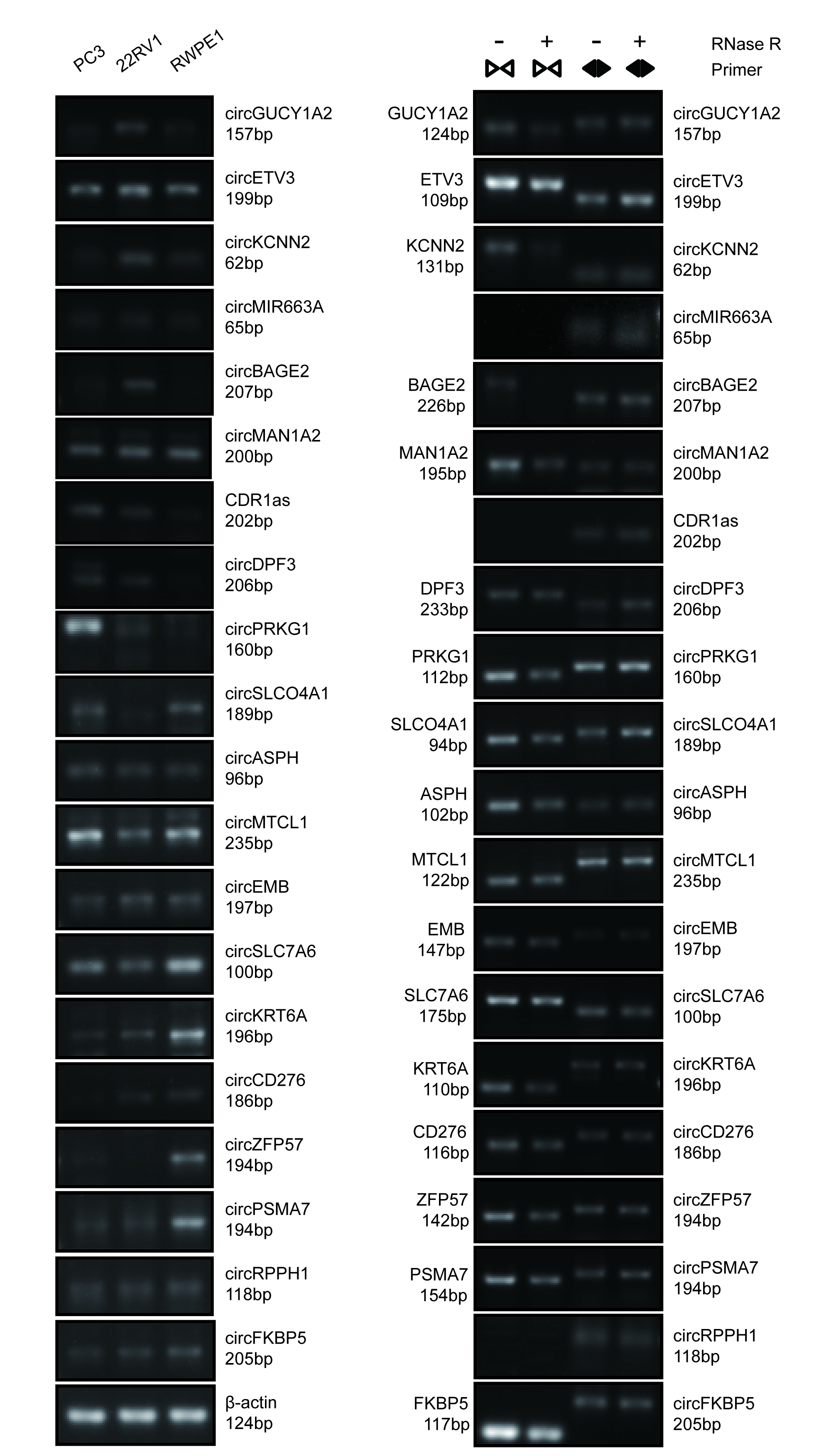


**Supplementary Figure S6**

**
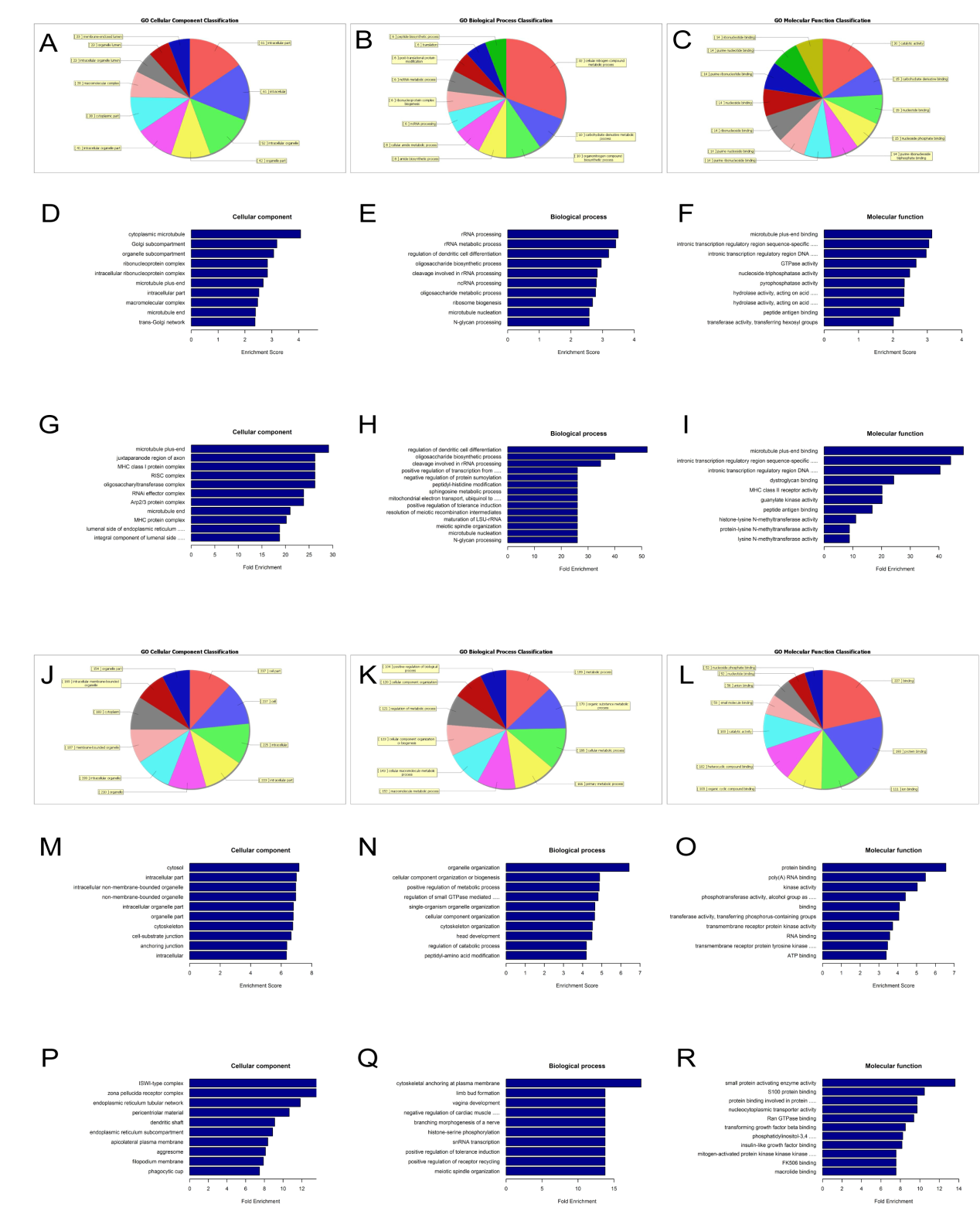
**

**Supplementary Figure S7**

**
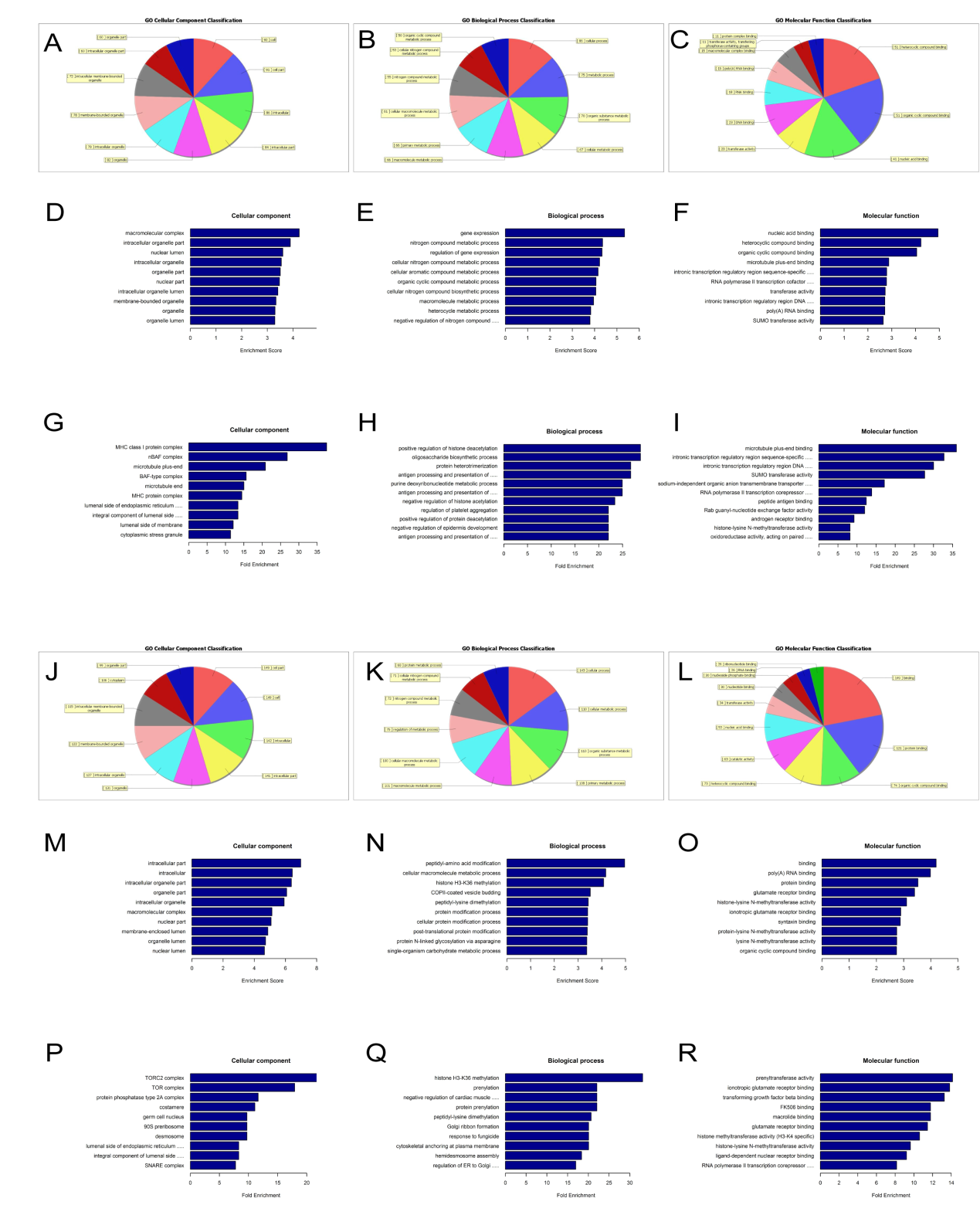
**

**Supplementary Figure S8**

**
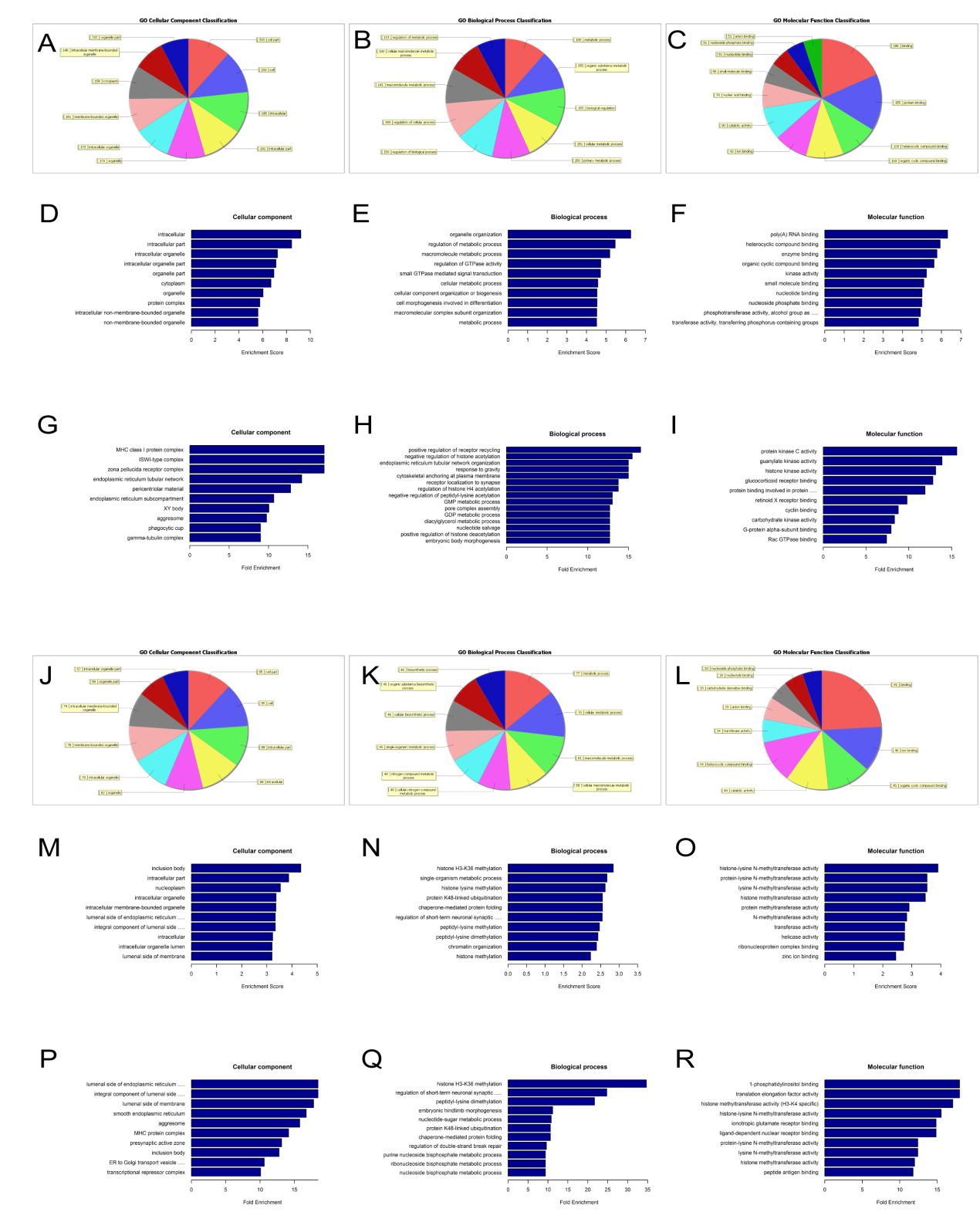
**

**Supplementary Figure S9**

**
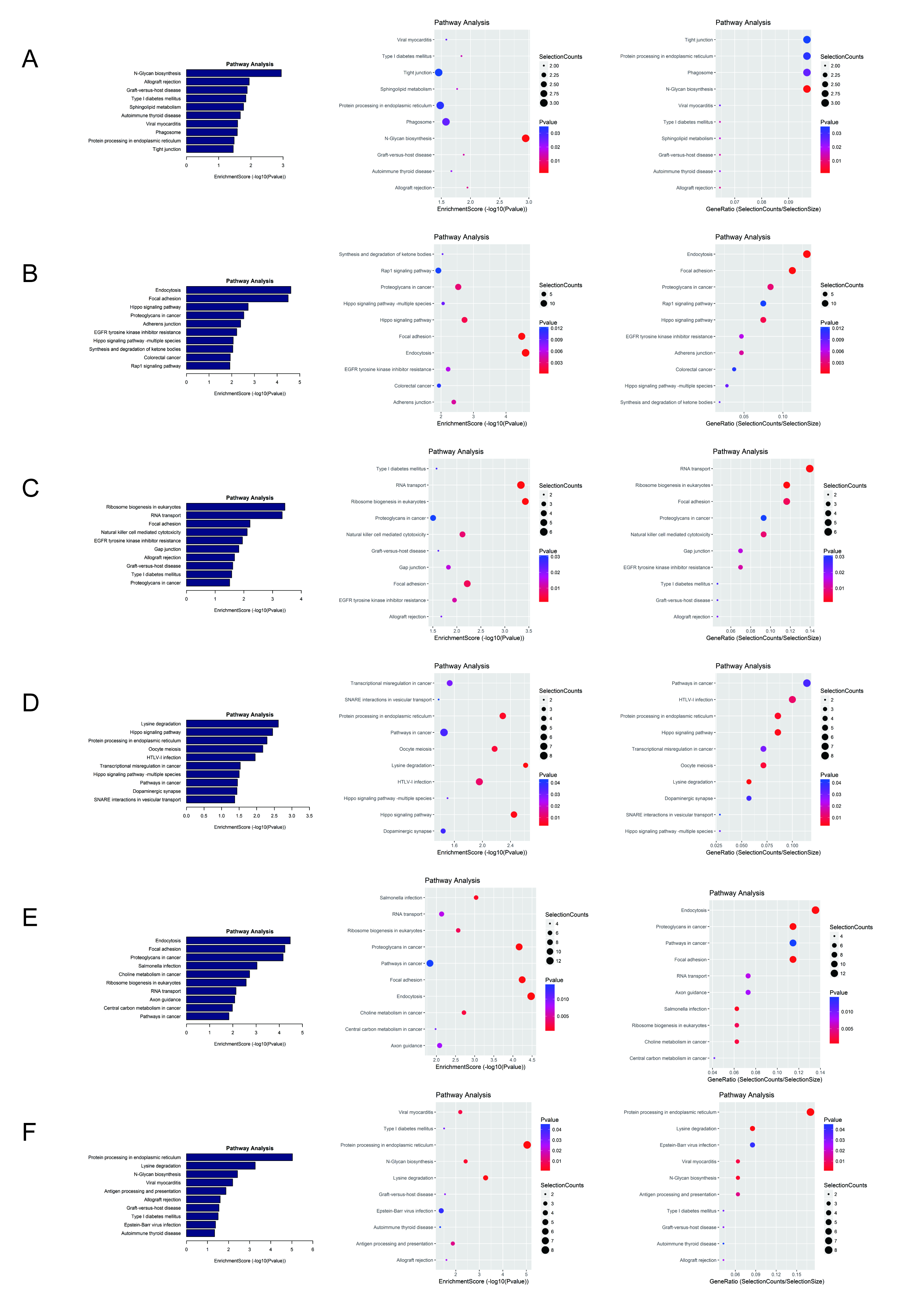
**

**Supplementary Table S1**

| **Sample** | **Raw reads** | **Mapped reads** | **Mapped percentage** |
| --- | --- | --- | --- |
| S1 | 25634600 | 24842045 | 97% |
| S2 | 21338001 | 20543097 | 96% |
| S3 | 18646467 | 18005214 | 97% |
| S4 | 38209299 | 35748812 | 94% |
| S5 | 18355309 | 17145195 | 93% |
| S6 | 21108304 | 19939701 | 94% |
| S7 | 20188166 | 19735663 | 98% |
| S8 | 23679547 | 23107721 | 98% |
| S9 | 22515364 | 21954214 | 98% |

**Supplementary Table S2 (shown in Differentially Expressed circRNAs.xls)**

**Supplementary Table S3**

| **Primer name** | **Primer sequence(5' - 3')-F** | **Primer sequence(5' - 3')-R** |
| --- | --- | --- |
| circ-GUCY1A2 | GCTCCTATGCAGACCACTCC | TTTCTGCATCCCTGTAACCA |
| GUCY1A2 | CGTGCTGGCTGGAGTTGTTG | TGGTAAGTGGTTGGGCTGACAT |
| circ-ETV3 | ACGGGGAATTTGTCATCAAG | AATGGGTAGTTGGGCATCAC |
| ETV3 | ACCAGTGCCAACAGCCTCTT | GCCAATCCCTCCTCCACCAAT |
| circ-KCNN2 | GGATAATTGCCGCATGGA | CTGCTCCATTGTCCACCA |
| KCNN2 | ACCATCAGGCAGCAGCAGAGA | GTTGGTGGTGCTGTGGAAGAGG |
| circ-MIR663A | CTACCGTTCTGCCTCCGA | CGCGTCTCGTCTCACTCA |
| circ-BAGE2 | AACTGGCATGGGTAAACCAG | TGTTCTGGACAAAGCAGGAA |
| BAGE2 | AATACAGTGAGCCCACCCTCGT | TTTCAGCTTTGACCTGCCTCGG |
| circ-MAN1A2 | GGAGGCCTACTTGCAGCATA | GCTTCTTCCAAGGCCTTCTC |
| MAN1A2 | GCGATTCACTCACGATCCAAGA | TCACCGGAGAACAGCAGATACA |
| CDR1as | CGTCTCCAGTGTGCTGATCT | GTCCGGAAGACATGGATTGT |
| circ-DPF3 | CCAAGCGAAAGAACAGGACT | GGCATCCACCTTCTTCTCAA |
| DPF3 | CTCTCAGGAAGACCACGACAA | CCAAGCAGAAGTCACAGTAGTT |
| circ-PRKG1 | GAGGGCTTTAACTGGGAAGG | AGCAGCATTTTTGGCAATCT |
| PRKG1 | GGACAGGACTCATCAAGCATAC | GTCTCTTCAAGGACATCAGCAA |
| circ-SLCO4A1 | GGAAATGCACCAGTTGAAGG | GGGTAACCAAGGATGGGAAC |
| SLCO4A1 | TGCCTCTGCCTCACCTTCGT | GCGTCCAACTCCACCTCATAGC |
| circ-ASPH | TGATGCTGATGGTGATGGA | CGCCTTTCCTCCCATTCT |
| ASPH | GAGCACGAGGTATGGCAGGATG | TGCTGGAAGGCTGCGTCTCT |
| circ-MTCL1 | AAAGCGCGCTAAAGCTGA | CTGCAGGATTCGGCAGTT |
| MTCL1 | AGCAGCAATATGCCAGCGACAA | CTCAGCGGAGGAGCCTAGTTCT |
| circ-EMB | TTCAGCTTTGTTTATCCTTCCTG | TGTTGTGAACTGGCATGTGA |
| EMB | AAGGTTCCTGTTGGTGTTCAA | TTCTTCACTCTCGCCTAATTGG |
| circ-SLC7A6 | TGGACCCATTTATGAGGAGGT | GCAAGGCCTAATTCCACTTG |
| SLC7A6 | GCCTATTACACAGTGCTGAACA | CCACGAAGAACAACCTTGATGA |
| circ-KRT6A | GCGTTGGACAAGTCAACATC | GAACTGAAGCCACCTCCAAC |
| KRT6A | TGGCGTTGGAGGTGGCTTCA | TGGAGGAGGAGGTGGTGGTGTA |
| circ-CD276 | AGCTTCACCTGCTTCGTGAG | ATCCTGCCAGAACACCTCAG |
| CD276 | CTCCCTACTCGAAGCCCAGCAT | TGCCAGAACACCTCAGCCTCAG |
| circ-ZFP57 | TGGCCAGAATCTTTCTGCAT | TCCTGGTAAAGGACCCTCTG |
| ZFP57 | CCAGAAGCCAGAGGTCCATCCA | CTGGTCACAGGTGCTTGGTTCT |
| circ-PSMA7 | CTCATCGTGGGTTTCGACTT | ATGCAGACGTTGTCATCCAA |
| PSMA7 | GCGGTTGGTGTTCGAGGAAGAG | CTATCCTTGCATCGGCGGTGAG |
| circ-RPPH1 | GGGCTCTCCCTGAGCTTC | CAAGGGACATGGGAGTGG |
| circ-FKBP5 | TCCCTCGAATGCAACTCTCT | TGGGGCTTTCTTCATTGTTC |
| FKBP5 | GCAGGCGGTGATTCAGTATGGG | GCCAGGTTCAGAAAGGCAGCAA |
| β-actin | CGCGAGAAGATGCCCAGATC | TCACCGGAGTCCATCACGA |
